# Supplementary material for: Direct tracking of H2 roaming reaction in real time
Source: Nat Commun. 2024 Aug 6;15:6656. doi: 10.1038/s41467-024-49671-6 (PMC11303762; doi:10.1038/s41467-024-49671-6)
Supplement: Supplementary file 1 — Supplementary Information [file 41467_2024_49671_MOESM1_ESM.pdf]

Supplementary Information – Direct tracking of H_2 roaming reaction in real time

Debadarshini Mishra*†,[1] Aaron C. LaForge*†,[1] Lauren M. Gorman,[1]
Sergio Díaz-Tendero,[2,3,4] Fernando Martín,[2,3,5] and Nora Berrah[1]

[1]*Department of Physics, University of Connecticut, Storrs, Connecticut, 06269, USA*
[2]*Departamento de Química, Módulo 13, Universidad Autónoma de Madrid, 28049 Madrid, Spain, EU*
[3]*Condensed Matter Physics Center (IFIMAC), Universidad Autónoma de Madrid, 28049 Madrid, Spain, EU*
[4]*Institute for Advanced Research in Chemical Sciences (IAdChem), Universidad Autónoma de Madrid, 28049 Madrid, Spain*
[5]*Instituto Madrileño de Estudios Avanzados en Nanociencia (IMDEA-Nano), Campus de Cantoblanco, 28049 Madrid, Spain, EU*

CONTENTS

I. Supplementary Note 1: Optical Setup 1

II. Supplementary Note 2: Reconstructed kinetic energy of D_2 neutral 2

III. Supplementary Note 3: $D_3{}^+$ formation via roaming neutral D 3

IV. Supplementary Note 4: Neutral Fragmentation Channel in 2-propanol 4

V. Supplementary Note 5: Timescales for $H_3{}^+$ and $D_3{}^+$ formation 6

VI. Supplementary Note 6: Simulation of electrostatic potential exerted on H_2 7

VII. Supplementary Note 7: Roaming H_2 dynamics 8

VIII. Supplementary Note 8: Additional data from experiment and simulations 10
- A. Internal excitation energy used in simulations 10
- B. Additional simulation figures 12
- C. Additional experimental figures 14

Supplementary References 16

References 16

I. SUPPLEMENTARY NOTE 1: OPTICAL SETUP

A schematic of our experimental setup is shown in Supplementary Figure 1. We use a 5 kHz Ti:Sapphire laser that produces 35 fs pulses with a central wavelength of 790 nm. The laser beam is split into two paths using a 50:50 beamsplitter. The pulses in both paths are independently compressed using grating compressors. The pump pulses travel along the path with fixed length, while the probe pulses are time-delayed with respect to the pump via a delay stage controlled by LabView software. The intensity of each path is finely controlled by a combination of a $\lambda/2$ waveplate and a polarizer. The beams are spatially and temporally overlapped using another 50:50 beamsplitter at the end of the optical table before being propagted into the COLTRIMS chamber.

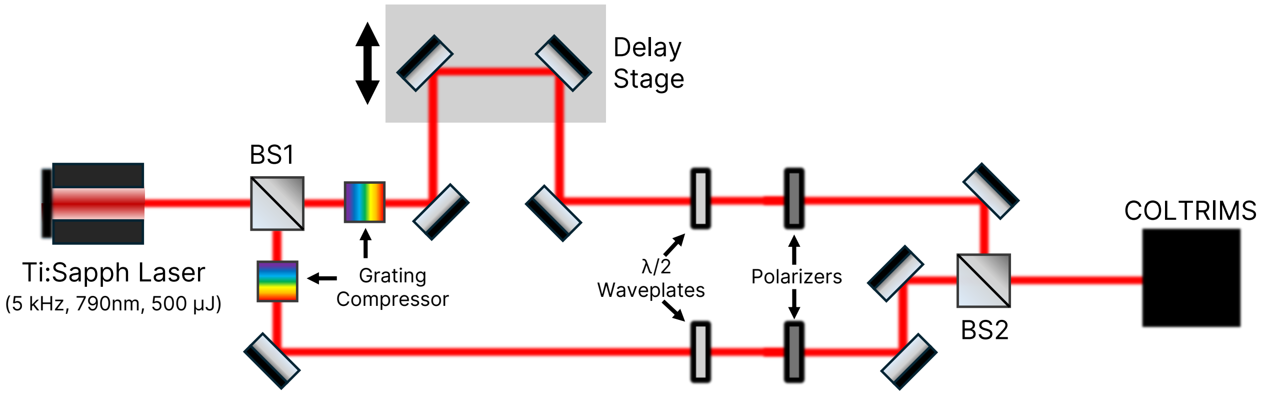

Supplementary Figure 1. A schematic of the experimental setup.

II. SUPPLEMENTARY NOTE 2: RECONSTRUCTED KINETIC ENERGY OF D_2 NEUTRAL

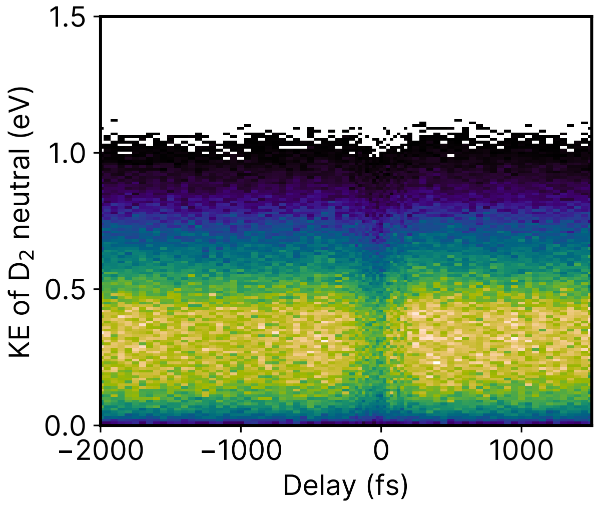

Supplementary Figure 2. Pump-probe delay dependent kinetic energy of the neutral D_2 from the channel D^+ + C_2N^+ + D_2.

To prove that the missing fragment D_2 in the incomplete channel, D^+ + C_2N^+, is indeed neutral, and not an ion that was simply lost in the detection process, we look at its kinetic energy as a function of pump-probe delay, shown in Supplementary Figure 2. The momentum vector and hence, the kinetic energy of D_2 are reconstructed by implementing momentum conservation among the three fragments. We observe a very low time-independent kinetic energy for D_2 which indicates that it is indeed a neutral dissociating from a charged fragment. On the contrary, the kinetic energy of an ion would typically decrease over time due to Coulomb explosion from the remaining charged moiety.

III. SUPPLEMENTARY NOTE 3: D_3^+ FORMATION VIA ROAMING NEUTRAL D

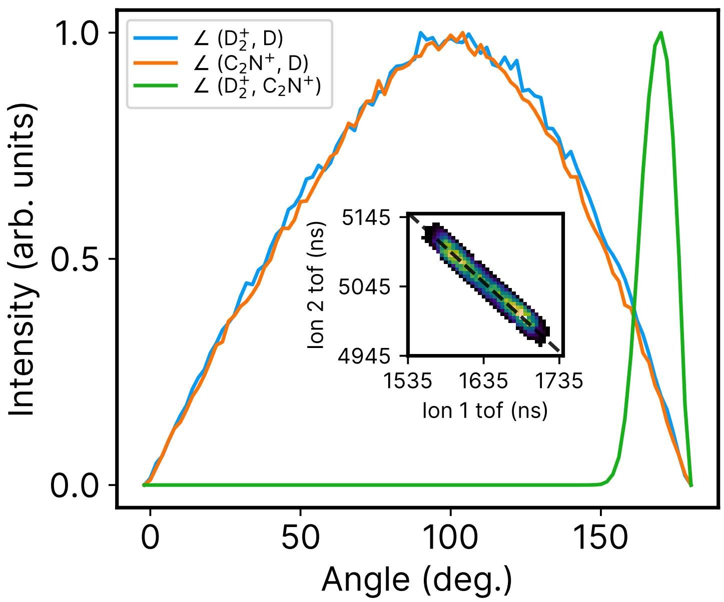

Supplementary Figure 3. Angular distributions between pairs of fragments in the incomplete D_2^+ + C_2N^+ + D channel

The fact that acetonitrile has only 3 hydrogen atoms that can contribute to D_3^+ formation makes it an ideal candidate to unambiguously track such roaming reactions. However, in order to be exhaustive in terms of all the possible ways that D_3^+ can be formed in acetonitrile, we must also consider the possibility of a roaming neutral D which can abstract D_2^+ from the remaining moiety to form D_3^+. For this, we have to consider the incomplete coincidence channel: D_2^+ + C_2N^+ + D. We note that the yield for this channel is nearly 4.5 times lower than D^+ + C_2N^+ + D_2, consequently making it a weak contributor to the D_3^+ channel. This is not surprising, since a free H or D atom is a radical, which makes it less likely to be formed than H_2 or D_2. In fact, this channel is not observed in our theoretical simulations due to the limited number of trajectories used in the present work. To our knowledge, it has also not been observed in previous works. Following the same sequence as before, we first ensure that the missing D is indeed a neutral by looking at its kinetic energy as a function of time-delay. Furthermore, in Supplementary Figure 3, we show the angular correlation between all pairs of fragments in the channel D_2^+ + D neutral + C_2N^+. The broad and symmetric angular correlations between the neutral D and the other two ionic fragments are indications of roaming D. Unlike in Fig. 4 (c) of the main article, the correlation between D and C_2N^+ is quite symmetric. This could be a result of D being a lighter roaming fragment compared to D_2.

IV. SUPPLEMENTARY NOTE 4: NEUTRAL FRAGMENTATION CHANNEL IN 2-PROPANOL

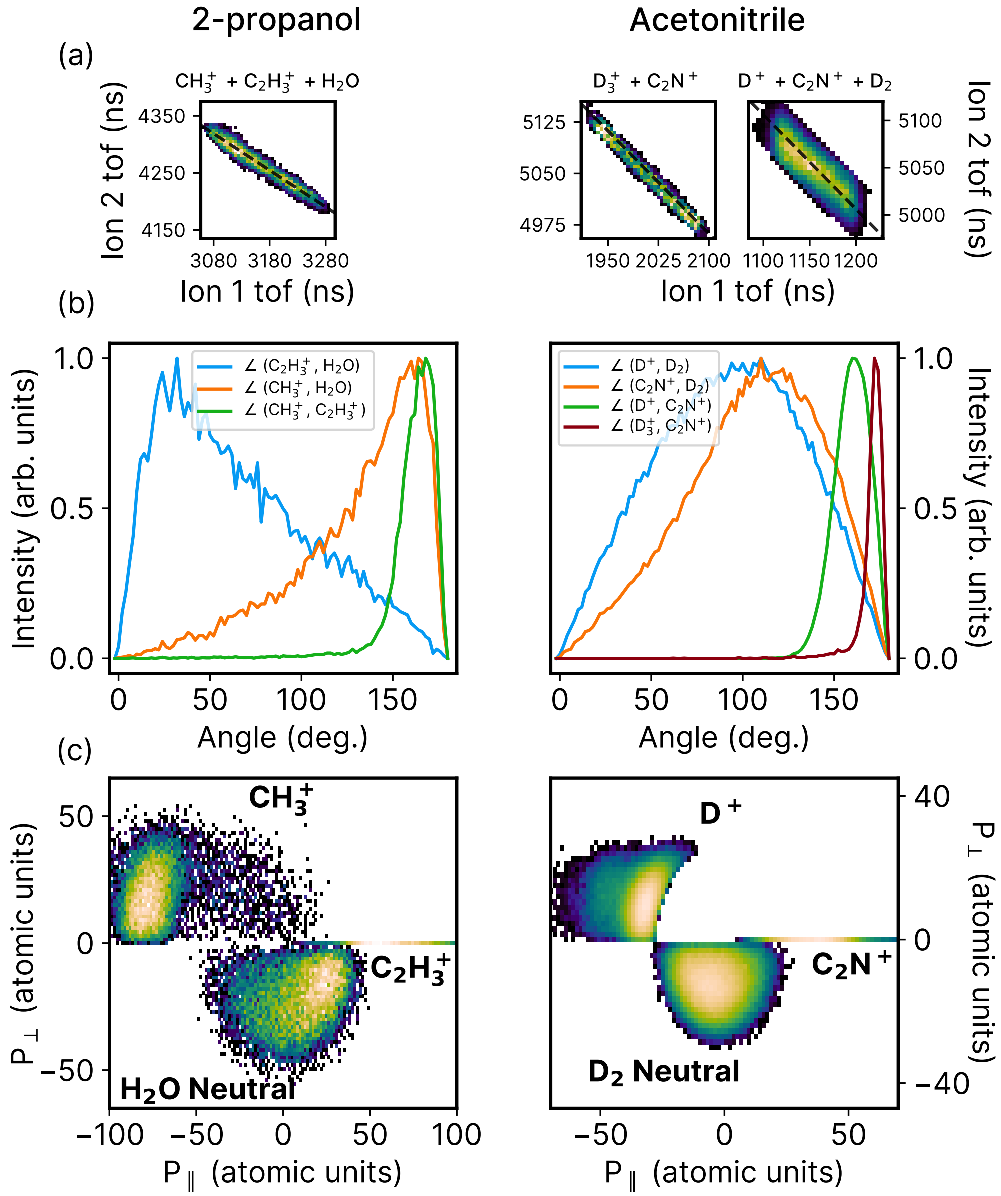

Supplementary Figure 4. (a) Incomplete PIPICO channel for $CH_3{}^+$ + H_2O neutral + $C_2H_3{}^+$ from 2-propanol integrated over the first 200 fs. A black dashed line of slope -0.643 is overlaid on the PIPICO channel to show the agreement between the calculated slope and experimental coincidence channel. (b) Angular distributions between the momentum vectors of $C_2H_3{}^+$ and H_2O (blue line), $CH_3{}^+$ and H_2O (orange line), $CH_3{}^+$ and $C_2H_3{}^+$ (green line) within the first 200 fs time-delay window. (c) Newton plot for the channel $CH_3{}^+$ + $C_2H_3{}^+$ + H_2O neutral integrated over the first 200 fs of pump-probe delay. The momentum vector of $C_2H_3{}^+$ lies along the x-axis while those of $CH_3{}^+$ and H_2O neutral are plotted in the top and bottom halves, respectively. Fig. 4 from the main article is shown in the right column for comparison to D_2 neutral roaming dynamics in acetonitrile.

In order to fully demonstrate the differences between a roaming and non-roaming neutral fragmentation channel, we have plotted in the left column of Supplementary Figure 4 the relevant dynamics from $CH_3{}^+$ + H_2O neutral + $C_2H_3{}^+$ in 2-propanol ($CH_3CHOHCH_3$). The PIPICO channel, shown in Supplementary Figure 4 (a), is for $CH_3{}^+$ measured in coincidence with $C_2H_3{}^+$, with a missing mass of H_2O. This channel involves two primary fragments, $CH_3{}^+$ and $C_2H_5O^+$, the latter of which subsequently dissociates into $C_2H_3{}^+$ and neutral H_2O, with a slope that can be calculated

as $-m_{C_2H_3^+}/(m_{C_2H_3^+} + m_{CH_3^+})$ = -0.643 [1]. A black dashed line of slope -0.643 is overlaid on this PIPICO channel to demonstrate the agreement between the expected and experimentally-obtained slope. Supplementary Figure 4 (b) shows the angular correlations between the momentum vectors of neutral H_2O and $C_2H_3^+$ (blue line) and neutral H_2O and CH_3^+ (orange line). Both these distributions are asymmetrically shifted either towards or away from 0° implying stronger momentum correlation, further corroborating that the neutral fragmentation of H_2O proceeds through a secondary decay from $C_2H_5O^+$. This behavior is in sharp contrast to that of corresponding angular distributions observed for the roaming neutral D_2 (shown to the right for comparison). Additionally, Supplementary Figure 4 (c) shows the Newton diagram for this channel over the first 200 fs pump-probe delay window. Here, the momentum vector of $C_2H_3^+$ is fixed along the x-axis, while those of CH_3^+ and the reconstructed H_2O are plotted on the top and bottom halves, respectively. Although the ionic fragments, $C_2H_3^+$ and CH_3^+, share similar characteristics to their counterparts in deuterated acetonitrile (shown in the right column of Supplementary Figure 4), the neutral fragments are significantly different. Specifically, the reconstructed momentum of neutral H_2O is strongly directed towards $C_2H_3^+$, which fully supports that H_2O is produced through a secondary decay from $C_2H_5O^+$. On the other hand, the reconstructed momentum of neutral D_2 displays no such preferential angularity and has a broad distribution centered at low momentum.

V. SUPPLEMENTARY NOTE 5: TIMESCALES FOR H_3^+ AND D_3^+ FORMATION

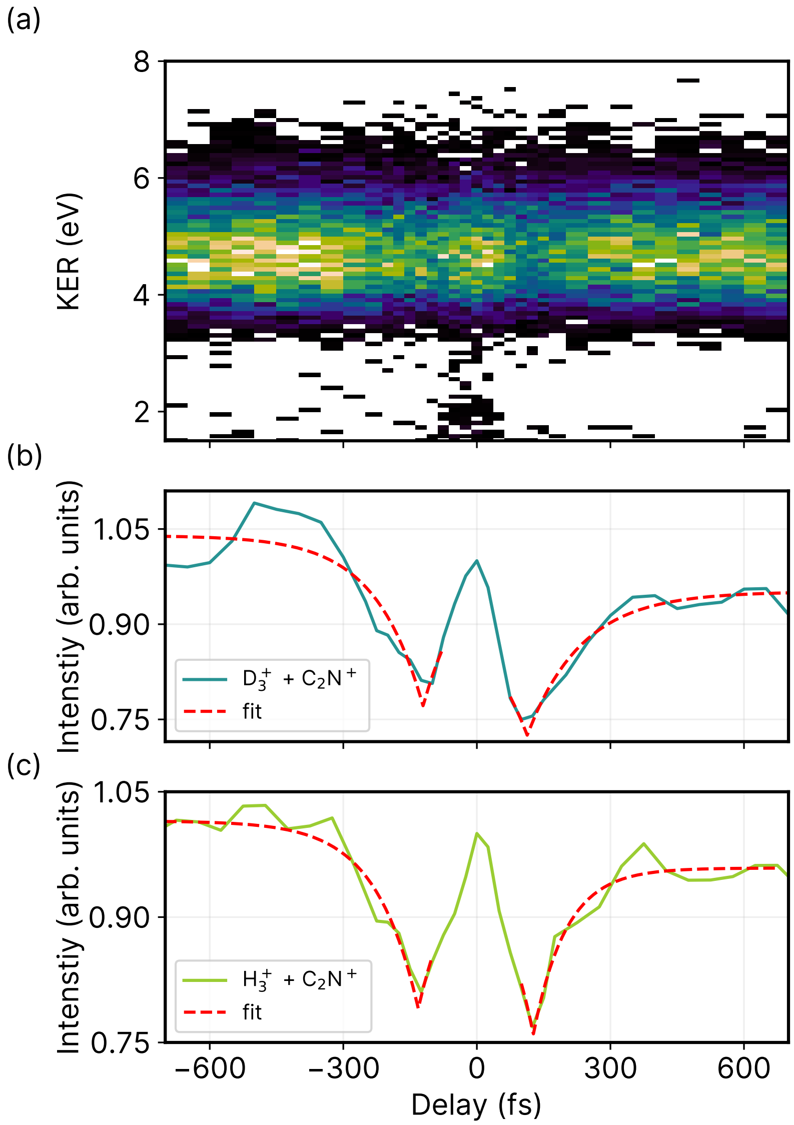

Supplementary Figure 5. (a) Kinetic energy release of $D_3^+ + C_2N^+$ as function of pump-probe delay. Projection of KER for (b) $D_3^+ + C_2N^+$ and (c) $H_3^+ + C_2N^+$ on the pump-probe delay axis. Red dashed lines obtained from the fit function (mentioned in the main text) are overlaid for comparison. The values from the fitting function are given in Supplementary Table 1.

**Signal**	$\mathbf{t}_0$	τ
H_3^+ Neg Delay	-131 ± 6 fs	111 ± 23 fs
H_3^+ Pos Delay	127 ± 6 fs	75 ± 20 fs
D_3^+ Neg Delay	-121 ± 10 fs	105 ± 30 fs
D_3^+ Pos Delay	113 ± 5 fs	122 ± 25 fs

Supplementary Table 1: Fit values

VI. SUPPLEMENTARY NOTE 6: SIMULATION OF ELECTROSTATIC POTENTIAL EXERTED ON H_2

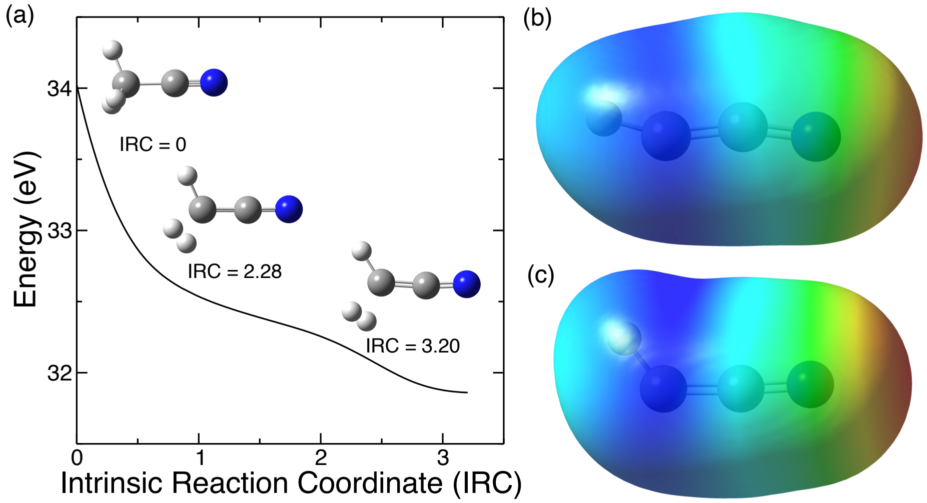

Supplementary Figure 6. (a) Minimum Energy Path (MEP) followed in the potential energy surface of the doubly ionized acetonitrile; relative energy in eV referred to the neutral molecule as a function of the Intrinsic Reaction Coordinate (IRC). (b) and (c) Electrostatic potential (ESP) of $HCCN^{2+}$ mapped on the electronic density with isovalue 0.0004 a.u.. Color code for the extreme values: red = 0.32 a.u. and dark blue = 0.48 a.u.. The geometry used in (b) corresponds to the channel $H_2/HCCN^{2+}$, i.e. after release of neutral H_2, and in (c) to the weakly bonded H_2...$HCCN^{2+}$, i.e. last point in the MEP.

Further insight into H_2 roaming has been obtained in a careful exploration of the potential energy surface (PES). In particular, we computed the minimum energy path (MEP) that a doubly charged acetonitrile molecule would follow after vertical ionization, i.e., the maximum gradient on the PES, starting from the optimized geometry of the neutral system (Supplementary Figure 6 (a)). We clearly observe the release of neutral H_2, followed by the production of a weakly bonded H_2...$HCCN^{2+}$ complex. Furthermore, this shows that the neutral H_2 is polarized by the potential exerted by the dicationic fragment. We evaluated such potential by computing the electrostatic potential (ESP) of $HCCN^{2+}$ in two different configurations: using the geometry of the optimized structure from the moiety (Supplementary Figure 6 (b)) and using the geometry of the last point given in the MEP exploration (Supplementary Figure 6 (c)). The ESP is plotted in both cases by projecting the actual value on the electronic density isosurface, which provides a visualization of the potential that polarizes the neutral H_2. Overall, Supplementary Figure 6 (b) and (c) reveal that the regions with the highest electrostatic potential (blue in color) are nearest to the C–H bond in $HCCN^{2+}$. It is most likely that the roaming H_2 remains weakly-bound in this region. Upon fragmentation of H^+ and CCN^+, the H_2 is approximately at 90° with respect to the two ionic fragments, which qualitatively agrees with the angular correlations given in Fig. 4 (d) of the main article.

VII. SUPPLEMENTARY NOTE 7: ROAMING H_2 DYNAMICS

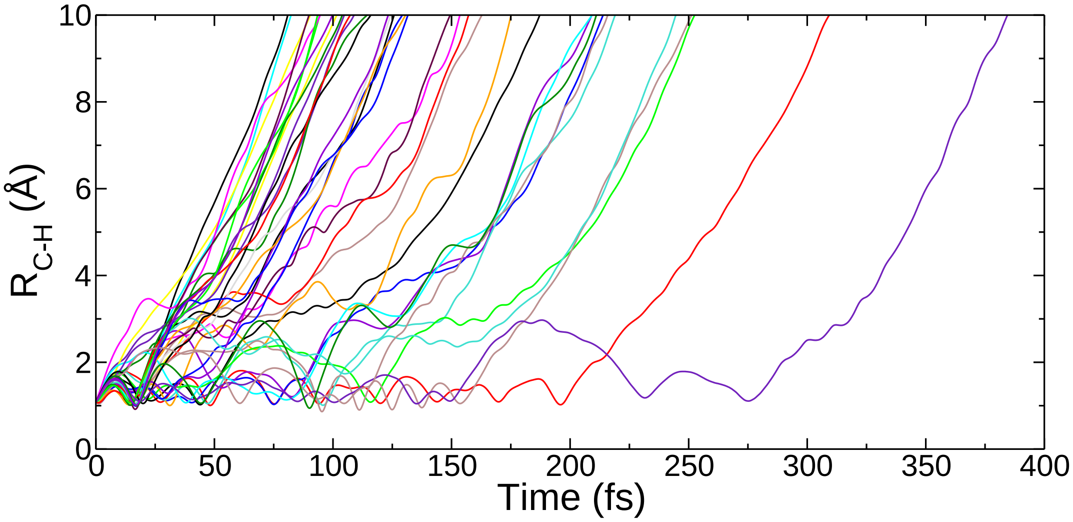

Supplementary Figure 7. Distance between the terminal carbon atom and one hydrogen atom of the emitted H_2 (in Å) as a function of time in the trajectories of the molecular dynamics simulations leading to H_2.

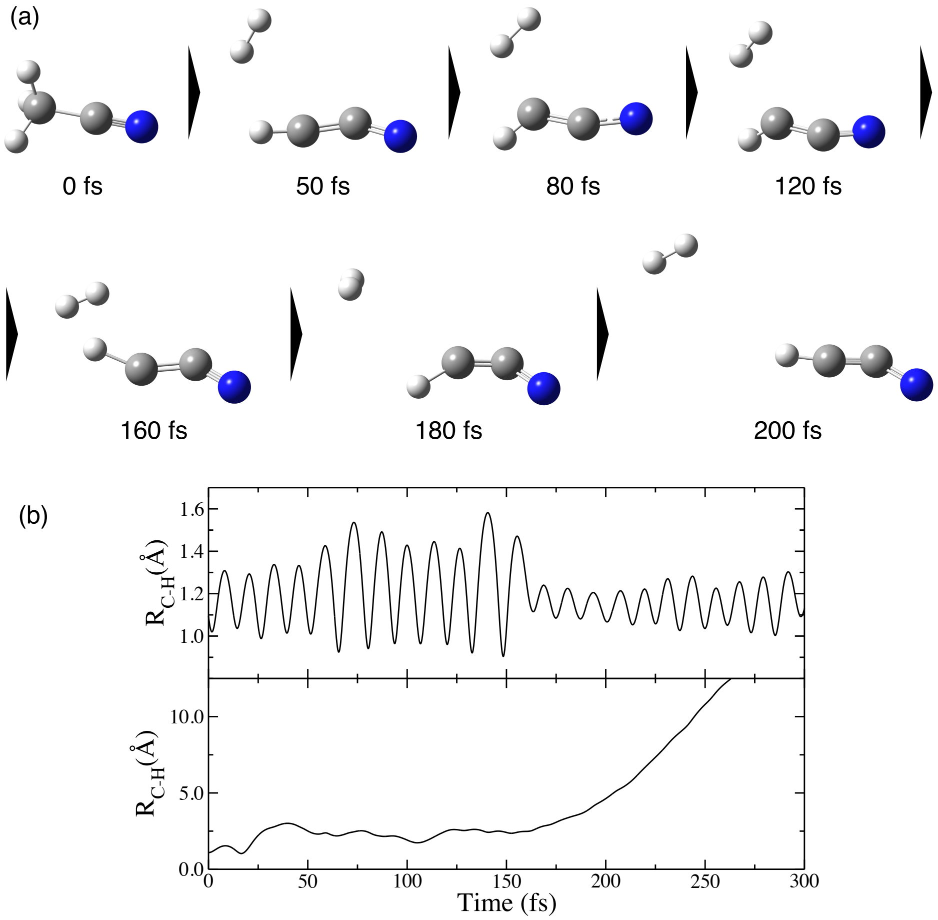

Supplementary Figure 8. Example of trajectory leading to H_2 emission. (a) Snapshots at different times. (b) Two C-H distances as a function of time: upper panel the H atom that remains bonded to the C atom ; lower panel one of the H atoms of the emitted H_2.

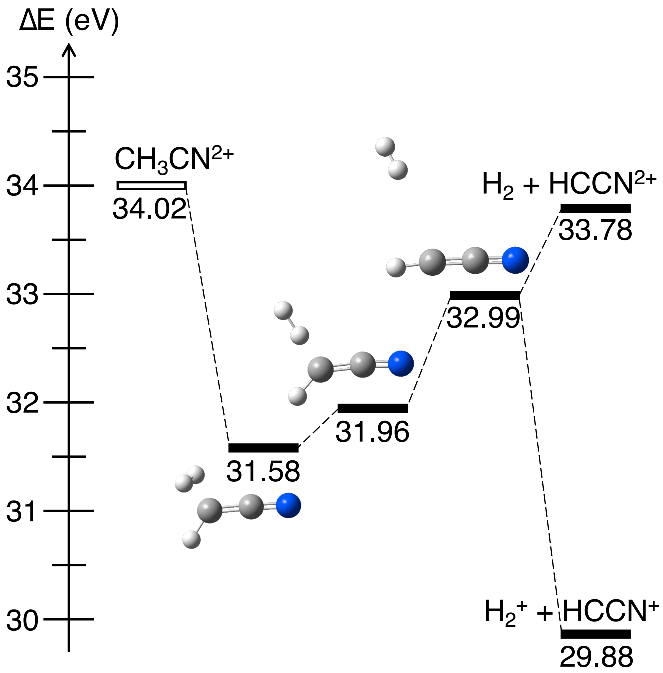

Supplementary Figure 9. Relevant stationary points in the potential energy surface of dicationic acetonitrile for H_2 emission. The open box corresponds to the vertical ionization from neutral acetonitrile (entrance channel). Relative energies are referred to neutral acetonitrile and those of the stationary points have been corrected with the zero-point-energy.

Among the 500 trajectories, we have identified 38 which lead to the release of H_2 (7.6% of trajectories). In Supplementary Figure 7, we show the H_2–HCCN distance as a function of time. The production of H_2 occurs in a time interval of 20 - 350 fs. In some of those cases, we clearly observe roaming trajectories. We have selected one of these trajectories and performed a detailed analysis (see Supplementary Figure 8 with snapshots and C-H distances). Here, we can see that the excitation energy is stored in C-H vibrational modes while the H_2 is roaming. At ∼180 fs the vibrational energy is transferred to the H_2, which is observed by the C-H vibrational amplitude decreasing while the kinetic energy of H_2 increases simultaneously. In other words, while H_2 is roaming, the C-H bond stores a significant amount of vibrational energy, and there is not enough translational kinetic energy to emit the H_2. As soon as the energy is transferred from the vibrational mode of the C-H bond to the translation of H_2, then emission occurs. It is impossible that an $H_2{}^+$ is the roamer since it would get expelled due to Coulomb repulsion. We can thus conclude that H_2 is produced after roaming. Supplementary Figure 9 shows the relevant stationary points in the potential energy surface where emission of H_2 is energetically accessible with a potential barrier. This is precisely the origin behind H_2 roaming before dissociation; the potential well keeps the H_2 weakly-bonded.

VIII. SUPPLEMENTARY NOTE 8: ADDITIONAL DATA FROM EXPERIMENT AND SIMULATIONS

Below are additional figures and data from simulations and experiment. See captions for more details.

A. Internal excitation energy used in simulations

Using an excitation energy of 3 eV in the molecular dynamics simulations gives very good agreement between the experimental branching ratios and those obtained from simulations. Supplementary Table 2 below presents the relative yields among three two-body coincidence channels of highest abundance, as observed in both experiment and simulations:

Channel	Exp. Yield	Rel. Exp. Yield	Theory Yield	Rel. Theory Yield
$H^+ + H_2C_2N^+$	$7,137,341$	90.32	398	90.45
$H_2{}^+ + HC_2N^+$	$736,730$	9.32	38	8.64
$H_3{}^+ + HC_2N^+$	$28,421$	0.36	4	0.91

Supplementary Table 2: Comparison of relative yields of the three most abundant two-body coincidence channels, as observed in both experiment and simulations with 3 eV internal excitation energy.

To confirm that one needs to run the simulations by introducing some amount of excitation energy, we have performed calculations for 500 trajectories in which the initial internal energy of the acetonitrile dication corresponds to the zero-point-energy of neutral acetonitrile, 1.23 eV. We are thus assuming ionization in the Frank-Condon region with a minimum amount of excitation energy. The results for the branching ratios are:

1 fragment (no fragmentation but possible isomerization) = 21.0%

$H^+/H_2C_2N^+ = 78.4\%$

$H_2/HC_2N^+ = 0.6\%$

These results do not explain the fragmentation observed in the experiment.

B. Additional simulation figures

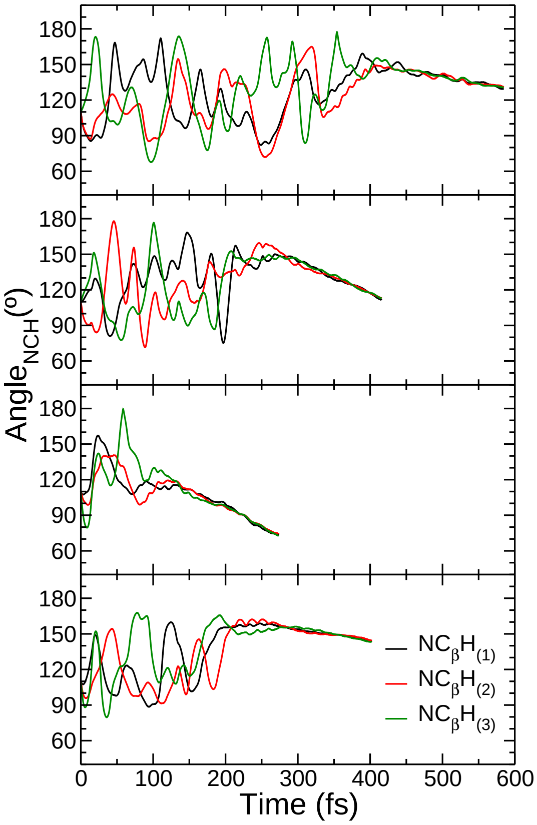

Supplementary Figure 10. Angles formed by the nitrogen, terminal carbon and hydrogen atoms (in degrees) as a function of time in the four trajectories of the molecular dynamics simulations leading to H_3^+. The angle for each hydrogen atom, $NC_\beta H_{(i)}$, is given in a different color for each trajectory. Movies of simulation results (snapshots for every 1 fs) of the four trajectories leading to H_3^+ formation are available as additional files.

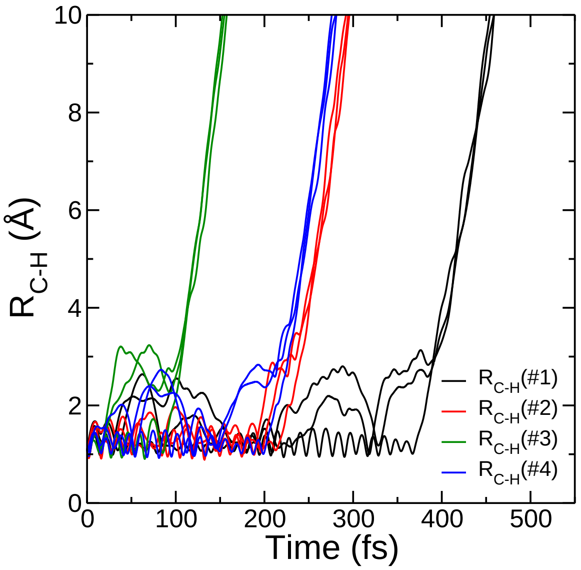

Supplementary Figure 11. Distance between the terminal carbon atom and each hydrogen atom (in Å) as a function of time in the four trajectories of the molecular dynamics simulations leading to H_3^+. The four trajectories, labelled (#i), are shown in different colors. Movies (snapshots at every 1 fs) of these 4 molecular dynamics trajectories leading to H_3^+ formation are included as Supplementary Movies. Movies of these four simulated molecular dynamics trajectories leading to H_3^+ formation, with snapshots at every 1 fs, are included as Supplementary files.

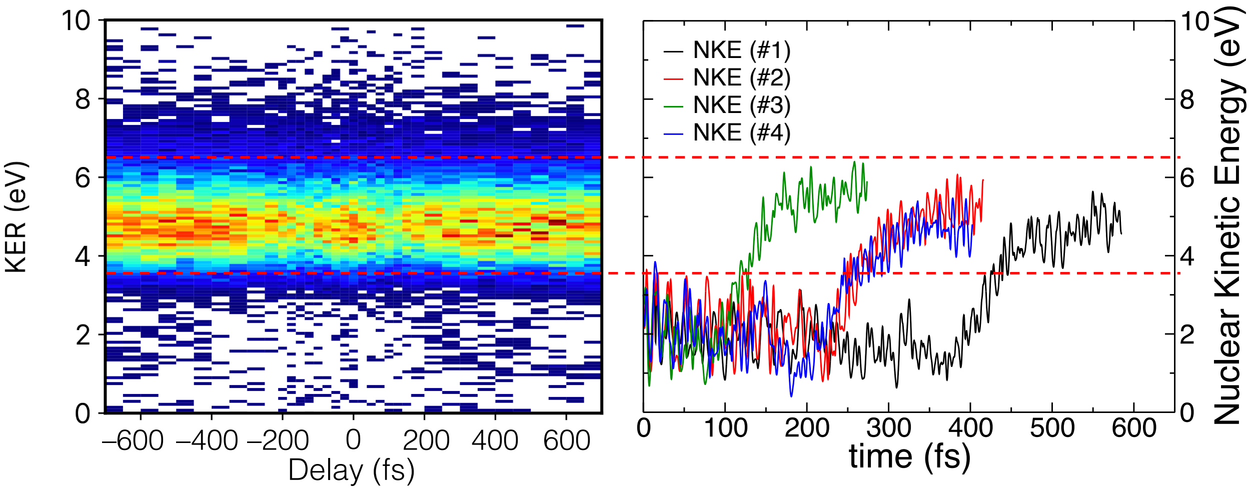

Supplementary Figure 12. Nuclear kinetic energy (NKE) as a function of time for the four trajectories leading to $H_3^+ + C_2N^+$, labelled (#i). Comparison with experimental KER shows good agreement.

C. Additional experimental figures

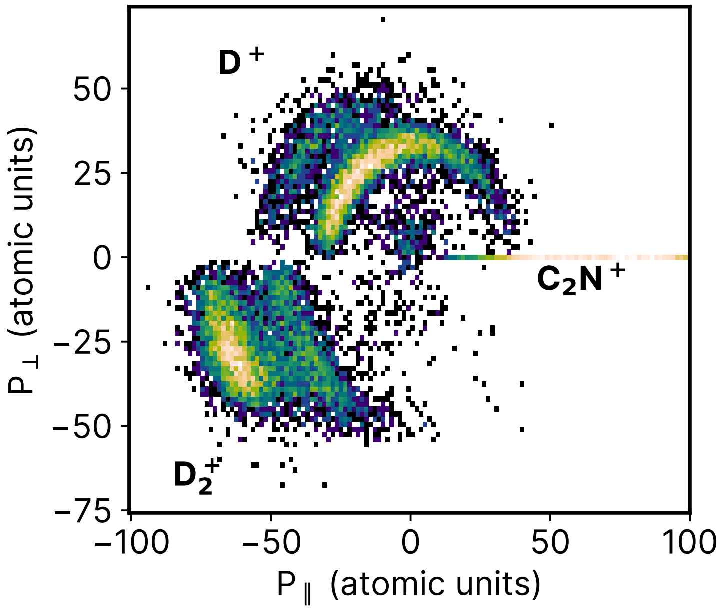

Supplementary Figure 13. Newton plots for the channel D^+ + $D_2{}^+$ + C_2N^+ integrated over 200 fs time-delay window. The momentum vector of C_2N^+ is fixed along the x-axis and the momentum vectors of D^+ and $D_2{}^+$ are plotted in the upper and lower halves of the plot, respectively.

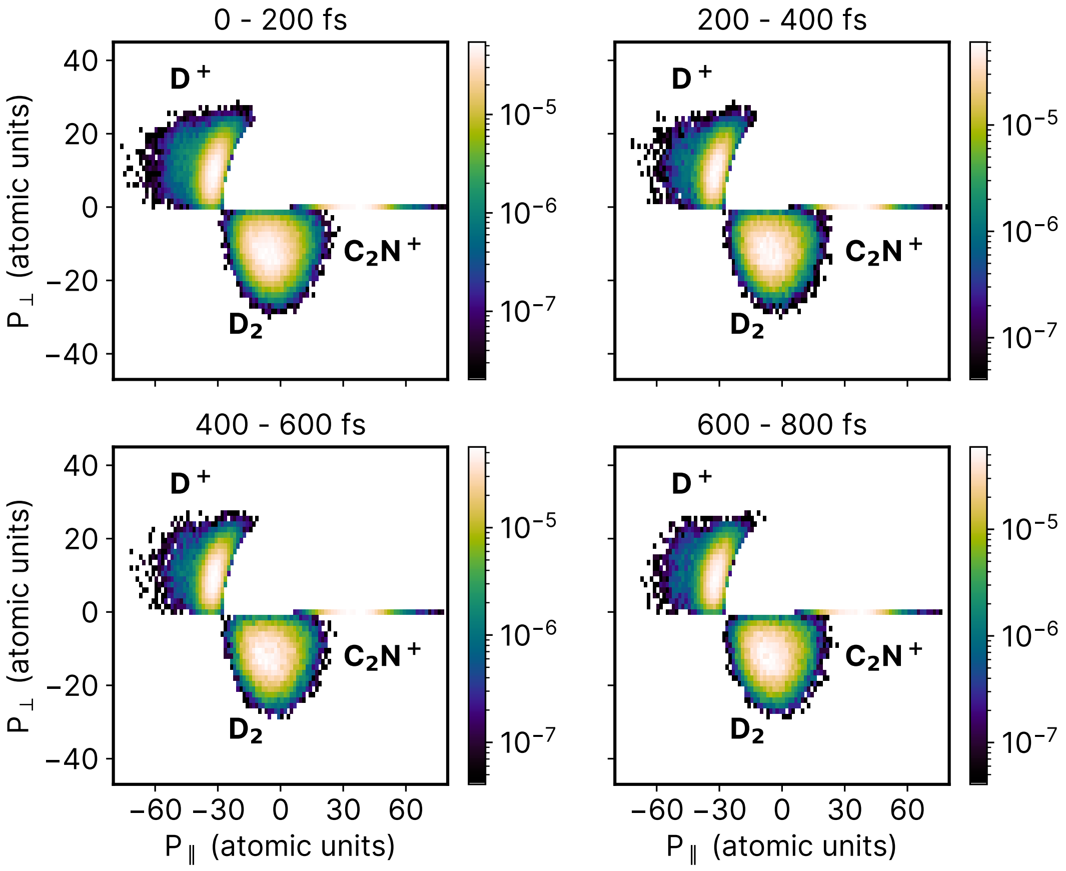

Supplementary Figure 14. Newton plots for the channel D^+ + D_2 + C_2N^+, integrated over 200 fs time-delay window, for four different time-windows: 0 – 200 fs, 200 – 400 fs, 400 – 600 fs, and 600 – 800 fs. The momentum vector of C_2N^+ is fixed along the x-axis and the momentum vectors of D^+ and D_2 are plotted in the upper and lower halves of the plot, respectively.

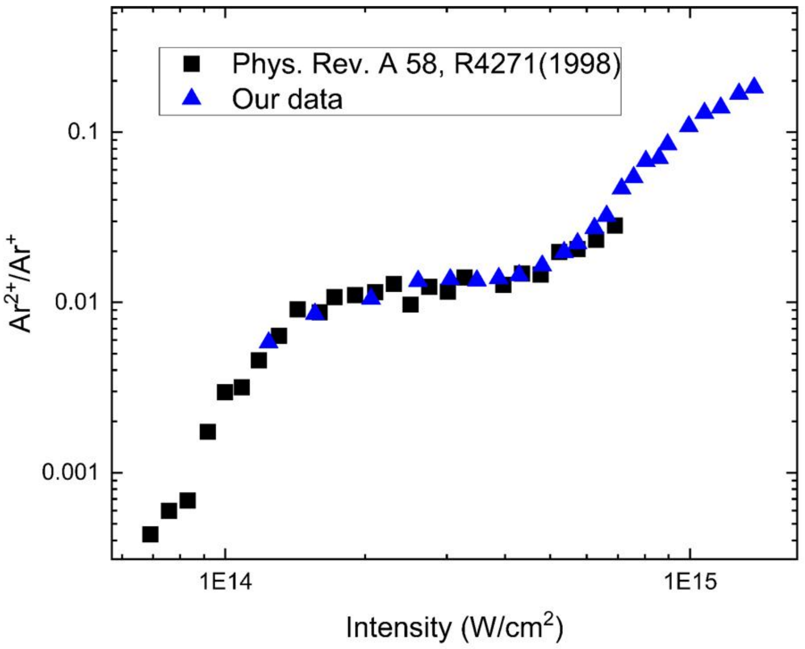

Supplementary Figure 15. Laser intensity calibration: ratio of double to single ionization yield in argon as a function of laser intensity. Our current data is given by blue triangles along with previously published results [2] given as black squares for comparison. Data is reproduced from [2] with permission from the American Physical Society ©1998.

SUPPLEMENTARY REFERENCES

[1] J. Eland, Laser Chem. **11**, 259 (1991).
[2] C. Guo, M. Li, J. P. Nibarger, and G. N. Gibson, Physical Review A **58**, R4271 (1998).

* contributed equally
† To whom correspondence should be addressed. Email: debadarshini.mishra@uconn.edu or aaron.laforge@uconn.edu
