# Supplementary material for: Direct tracking of H2 roaming reaction in real time
Source: Nat Commun. 2024 Aug 6;15:6656. doi: 10.1038/s41467-024-49671-6 (PMC11303762; doi:10.1038/s41467-024-49671-6)
Supplement: Supplementary file 3 — Description of Additional Supplementary Files [file 41467_2024_49671_MOESM3_ESM.pdf]

Supplementary Movie 1: Movie showing 1 fs snapshots of a typical molecular dynamics trajectory leading to the formation of H_3^+ in doubly-ionized acetonitrile.

Supplementary Movie 2: Movie showing 1 fs snapshots of a typical molecular dynamics trajectory leading to the formation of H_3^+ in doubly-ionized acetonitrile.

Supplementary Movie 3: Movie showing 1 fs snapshots of a typical molecular dynamics trajectory leading to the formation of H_3^+ in doubly-ionized acetonitrile.

Supplementary Movie 4: Movie showing 1 fs snapshots of a typical molecular dynamics trajectory leading to the formation of H_3^+ in doubly-ionized acetonitrile.
