# Supplementary figures and images for: Direct tracking of H2 roaming reaction in real time
Source: Nat Commun. 2024 Aug 6;15:6656. doi: 10.1038/s41467-024-49671-6 (PMC11303762; doi:10.1038/s41467-024-49671-6)

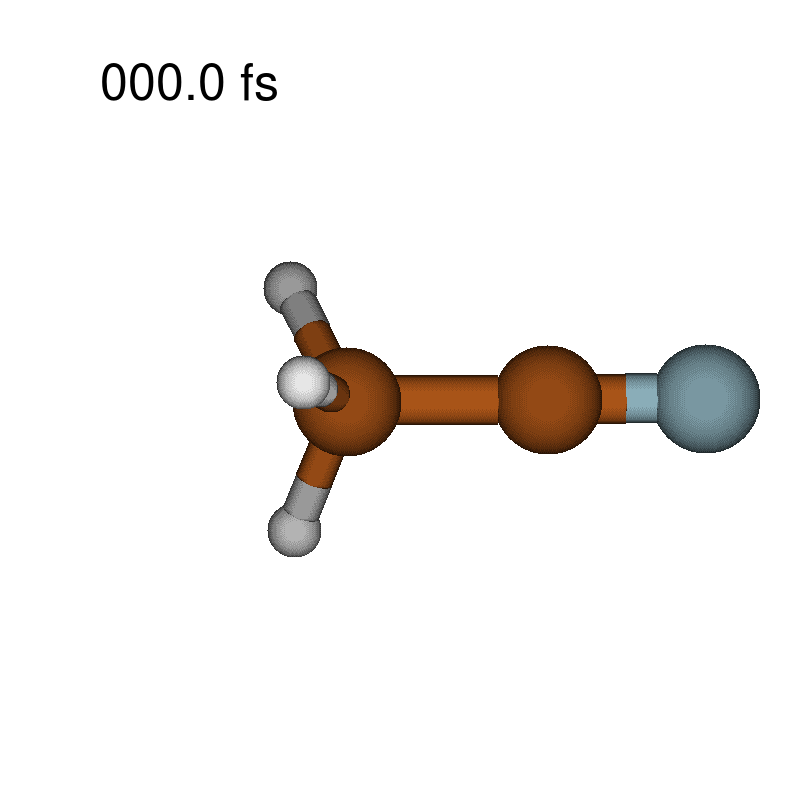

Supplement: Supplementary file 4 — Supplementary Movie 1 [file 41467_2024_49671_MOESM4_ESM.gif]

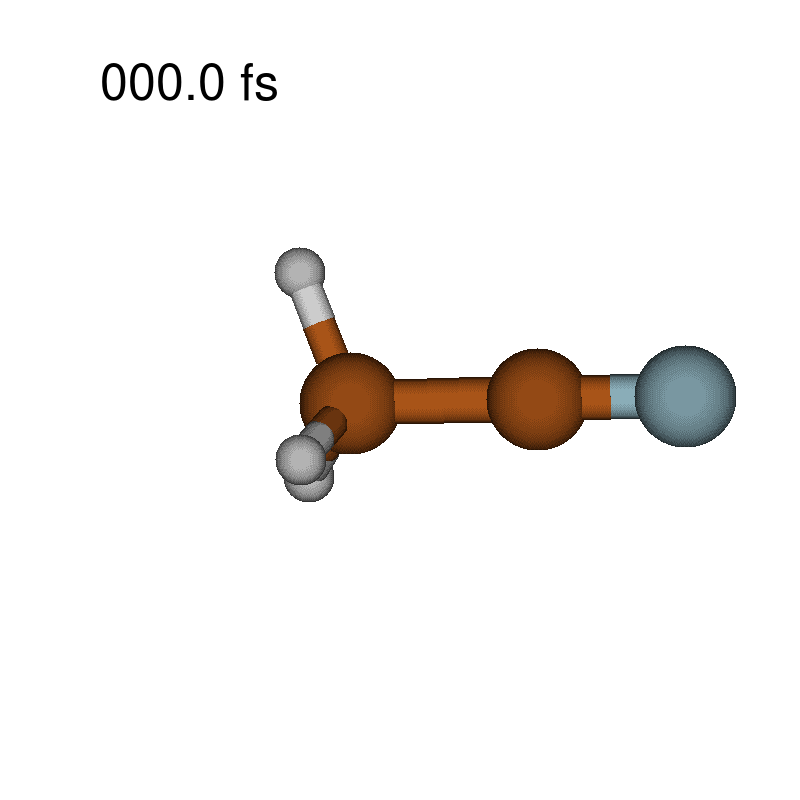

Supplement: Supplementary file 5 — Supplementary Movie 2 [file 41467_2024_49671_MOESM5_ESM.gif]

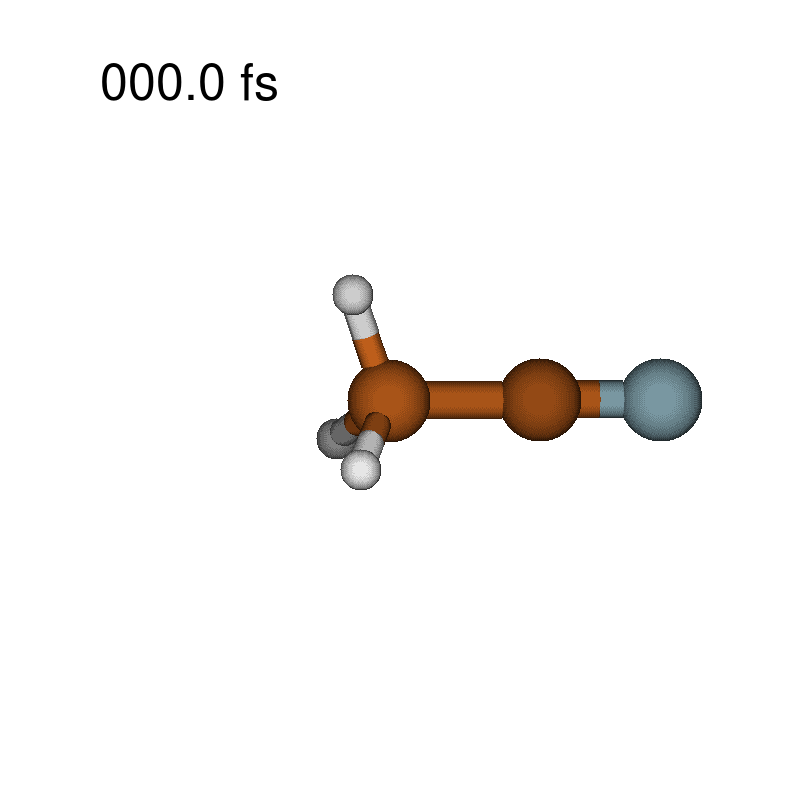

Supplement: Supplementary file 6 — Supplementary Movie 3 [file 41467_2024_49671_MOESM6_ESM.gif]

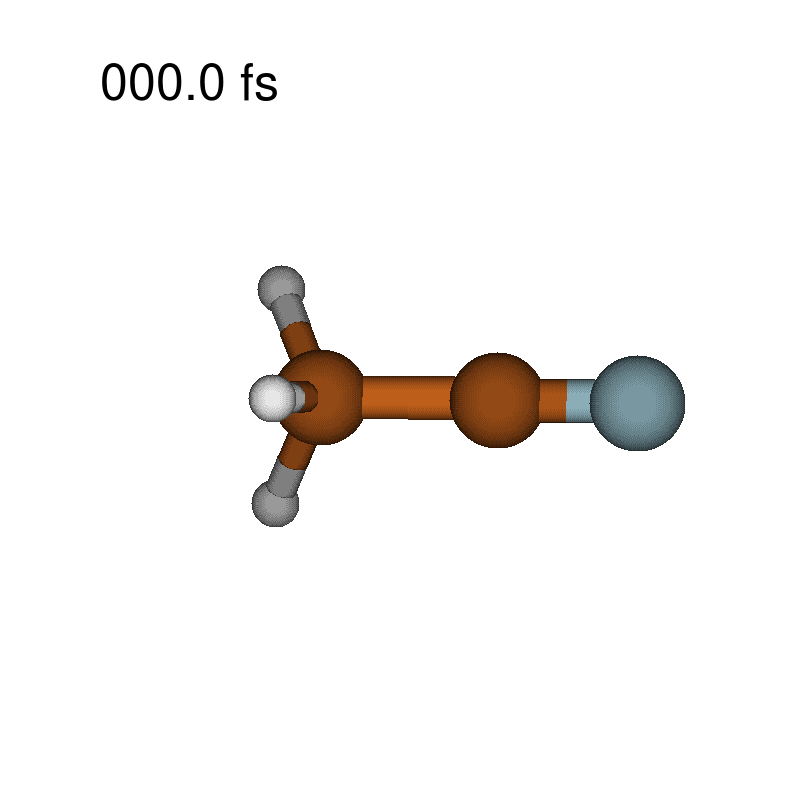

Supplement: Supplementary file 7 — Supplementary Movie 4 [file 41467_2024_49671_MOESM7_ESM.gif]
